# Adsorption Properties of Essential Oils on Polylactic Acid Microparticles of Different Sizes

**DOI:** 10.3390/ma15196602

**Published:** 2022-09-23

**Authors:** Lilla Virág, Róbert Bocsi, Dóra Pethő

**Affiliations:** Research Centre for Biochemical, Environmental and Chemical Engineering, University of Pannonia, Egyetem u. 10, 8200 Veszprém, Hungary

**Keywords:** polylactic acid microparticles, Hansen solubility parameter, essential oil adsorption

## Abstract

The interaction between the polymer and the materials in contact with it affects its applicability. This can be particularly important in applications such as packaging or controlled drug delivery systems. Because of these interactions, the adsorption and diffusion properties of polylactic acid (*PLA*) are important. The absorption capacity of different polylactic acid particles for different additives like essential oils (*Thymus vulgaris*, *Melissa officinalis*, and *Foeniculum vulgare* essential oils) was investigated depending on the concentration of the essential oil. The *PLA* microparticles were prepared by the solvent evaporation emulsification method. The prepared particles had a degree of crystallinity of 0.1% and 16.1%, respectively, according to the granules used. This affects the particles’ adsorption properties. The specific essential oil uptake of the more crystalline microparticles was on average 15% higher than that of the amorphous particles. The specific amount of essential oil adsorbed decreases with the decreasing concentration of essential oil in the solutions. We also investigated whether the amount of essential oil taken up was correlated with the solubility parameter of the essential oils. We concluded that the difference between the adsorption of the essential oils on the polymer was related to the essential oils’ Hansen solubility parameter.

## 1. Introduction

Polylactic Acid (*PLA*) is a biodegradable polymer widely used in medical implants and devices, tissue engineering, fibers for packaging containers, and textiles [1,2,3,4]. Certain properties, such as the low mechanical strength of the polymer, limit its applications. Essential oils can be used to modify these properties of *PLA* [1,2,3]. Essential oils and vegetable oils plasticize the polymer, reducing its tensile strength and increasing its elongation at break. The reason for the decrease in tensile strength is that the essential oil components weaken the interactions between the polymer molecules, increasing the flexibility of the polymer chains [5,6,7,8]. The thermal properties of polymer composites are also affected by essential oils. The glass transition temperature, melting temperature, and crystallization temperature are reduced for *PLA* composites containing essential oils [6,7,8].

The study of the adsorption and diffusion properties of essential oils in relation to biodegradable polymers (poly(lactic acid), poly(lactide-co glycolide)) is important in order to increase the applicability of the polymer. Essential oils, like lemongrass, thyme, or fennel essential oils are complex mixtures of different compounds, mainly terpenoids (citral, geranyl acetate, anethol, thymol, carvacrol). The applications of essential oils include: pharmaceuticals, cosmetics (creams, cleaning agents), the food industry (food preservatives as active packing material), and agricultural applications (insecticides, pesticides) [9,10,11,12].

*PLA*’s adsorption of the essential oils and its key components are affected, for example, by the applied impregnation method, the type of essential oil, and the key components’ polarity. The impregnation of the essential oil into the *PLA* matrix can be achieved on the one hand by encapsulation using the solvent evaporation emulsion method during the preparation of microparticles [12]. 

Martins et. al. examined the addition of thyme essential oil by encapsulation. The thyme essential oil contains a high percentage of phenolic polar compounds (62.2%), among which thymol prevails (47.7%). During the preparation of microcapsules, a high encapsulation efficiency, ~65%, was achieved by Martins et. al. The encapsulation efficiency shows the percentage of the essential oil that is incorporated into the *PLA* microcapsules. It was 80% for the apolar compounds, while for the polar compounds it was only 54% [13]. The authors confirmed that encapsulation is more favorable for apolar compounds than for polar components [14,15].

Fraj et. al. impregnated *PLA* microparticles in a similar way, with ~40% encapsulation efficiency [9]. The weight ratio of the essential oil and the polymer also has an effect on the efficiency. Oregano essential oil has been encapsulated in chitosan nanoparticles by Hosseini et. al. The authors found that by increasing the weight ratio of oregano essential oil to the chitosan, the encapsulation efficiency is decreased [16].

Essential oils’ impregnation into the *PLA* can be achieved by supercritical impregnation using supercritical CO_2_. The amount of impregnated essential oil may vary depending on what parameters (temperature, pressure, and depressurization rate) were used during the process. For example, during the impregnation of thymol, Torres et al. were able to impregnate the component in *PLA* films with 18–20% efficiency. The authors investigated the effect of the process parameters, e.g., pressure and depressurization rate, on the amount of the impregnated thymol [17]. In a study by Vileas et al., cinnamaldehyde was impregnated with 9–13% impregnation efficiency using similar parameters to Torres et. al., and Vileas et. al. found that slow depressurization rate and higher pressure favor the impregnation of the active component into the *PLA* matrix [17,18].

Martins et. al., Biswal et. al., and Dusankova et. al. investigated the release mechanism of essential oil components from *PLA* microspheres [13,19,20,21]. Martins et. al. investigated the release of thymol and p-cymol from *PLA*-based microcapsules [19]. They observed that due to diffusion through the *PLA* matrix in the first hours, the rate of release was higher for both components. After the first hours, the active component release rate was set to a constant value. They observed that the release mechanism after the first hours depends mainly on the molar mass of the *PLA*. The diffusion of the essential oil components is faster when the polymer molar mass is lower, because the mobility of the *PLA* chains is reduced if the molar mass of the polymer is higher. The diffusion of thymol occurs more rapidly through the polymer, probably due to the difference in polarity between the two components. They found that the diffusion coefficient was 1.99 × 10^−16^ m^2^/s for thymol and 4.34 × 10^−16^ m^2^/s for p-cymene in the first hour of release [13,19]. Dusankova et. al. have studied the preparation and characterization of *PLA* microspheres containing essential oil components of different polarities. The polar essential oil component is more adsorbed in the *PLA* microspheres than the more apolar components [21].

Martins et. al., Biswal et. al., and Dusankova et. al. have found that the change in the adsorption properties of the polymer can be associated with the properties of the essential oil components [13,19,20,21]. Based on this, it should be examined whether the Hansen solubility parameter (*HSP*) of essential oil components or the *HSP* of the solvents correlates with their adsorption properties to the polymer. The Hansen solubility parameter characterizes the affinity of the polymer for different organic solvents or components [22,23]. The *HSP* can be divided into three parameters: the parameter indicating the contribution to dispersion forces (*δ_d_*), the parameter for polar interactions (*δ_p_*), and the parameter representing the H-bonds formation (*δ_h_*). If the *HSP* of the organic solvents is close to the *HSP* of the polymer, the solvent is considered compatible with the polymeric material [24,25].

In our study, we investigated the adsorption properties of different polylactic acid particles for different additives such as essential oils. The essential oil uptake of *PLA* particles as a function of essential oil concentration and the solubility parameter of essential oils was investigated. The experiment was performed with three different essential oil solutions, using three different essential oil concentrations (0.25 mg/mL, 0.50 mg/mL, and 1.00 mg/mL), and two solvents (ethanol and methanol).

The microparticles were prepared by the solvent evaporation emulsion method [26,27,28]. In the single emulsion method, the organic phase containing the polymer is emulsified in an aqueous phase containing a stabilizer. Evaporation of the organic solvent leads to the hardening of the microspheres [29]. In the solvent evaporation emulsion method, the concentration of *PLA*, the amount of surfactant, the ratio of the aqueous phase to the organic phase, and the mixing rate play an important part in the properties of the microparticles [29]. For example, the concentration of the *PLA* solution affects the particle size, while the type of organic solvent used for the *PLA* solution affects the structure of the particles [26,28,29]. The amount and type of surfactant and the composition of the aqueous phase (e.g., the presence of NaCl) influence the microparticle solidification time [26,30]. We have carried out some preliminary experiments (which are not included in this report) in order to find the parameters that will allow us to produce suitable particles for our experiments.

## 2. Materials and Methods

Two different types of *PLA* granules have been used: NatureWorks Ingeo Biopolymer 4043D, and NatureWorks Ingeo Biopolymer 3D850 (Plymouth, MN, USA). The 3D850 granules were developed for manufacturing 3D printer monofilaments (henceforth referred to as “*F*”), while the 4043D granules were developed for the preparation of biaxially oriented films (henceforth referred to as “*P*”) [31,32]. Three kinds of essential oils were used for the experiment: *Melissa officinalis* (lemongrass essential oil, Neuston Healthcare Kft.), *Foeniculum* vulgare (fennel essential oil, Neuston Healthcare Kft., Budapest, Hungary), and *Thymus vulgaris* (thyme essential oil, Neuston Healthcare Kft.).

The microparticles were prepared based on a solvent evaporation emulsion method used by Zang [27]. The particles were prepared as follows: first, we prepared 100 mL *PLA* solution at a given concentration (5.0 wt.%) using dichloromethane solvent; then, the solution was added to 200 mL of 1 wt.% polyvinyl-alcohol (*PVA*) aqueous solution. The emulsion was stirred at a constant stirring speed (magnetic stirrer, 650 rpm) for 24 h, then after filtration and washing, the solid particles were dried in an oven at 50 °C for 24 h (Binder FD 53, Sigma, St. Louis, MO, USA).

For the determination of average diameter and size distribution of the particles, microscopic pictures (zoom: 40×) of the fractions were taken using an optical microscope (Lacerta, Wien, Austria). The particle size was determined from the images using a program (ImageJ, National Institutes of Health, Bethesda, MD, USA).

For the adsorption measurement, 1.000 g of *PLA* particles (in the case of the measurements with porous particles, 0.200 g) were weighed on an analytical balance (Ohaus Adventurer AR3130, Parsippany, NJ, USA) in a pre-weighed dry test tube, then 2.000 g of essential oil solution was added to it in a given concentration (0.25 mg/mL, 0.50 mg/mL, and 1.00 mg/mL). The microparticles were soaked for 24 h, then the samples were separated by filtration. 

The essential oil concentration of the residual solutions was analyzed by UV-Vis spectrophotometry. The absorbance spectra of the samples were taken between 200 and 800 nm with an Agilent Cary 60 UV-VIS Spectrophotometer (Santa Clara, CA, USA). 

Differential scanning calorimetry (*DSC*) was performed with NETZSCH DSC 214 Polyma instrument. The measurements were carried out under 60 mL/min N_2_ flow rate according to the following protocol: first and second heating from 20 to 200 °C with 10 °C/min heating rate, and a first cooling (after first heating) from 200 to 20 °C with 10 °C/min cooling rate.

For the investigation of how the solvent uptake is related to the Hansen solubility parameter (*δ_t_*) of essential oils, we determined the essential oils solubility parameter. The *δ_t_* and its components (*δ_d_*, *δ_p_*, *δ_h_*) were calculated using the Hoftyzer–Van Krevele method [24,33].

In this study, a three-letter notation was applied to the samples. The first letter refers to the type of particles, the second refers to the type of solvent and the last letter refers to the essential oil. The number refers to the concentration of the essential oil solution (Table 1). 

## 3. Results and Discussion

### 3.1. Microparticles Properties

**Figure 1:**
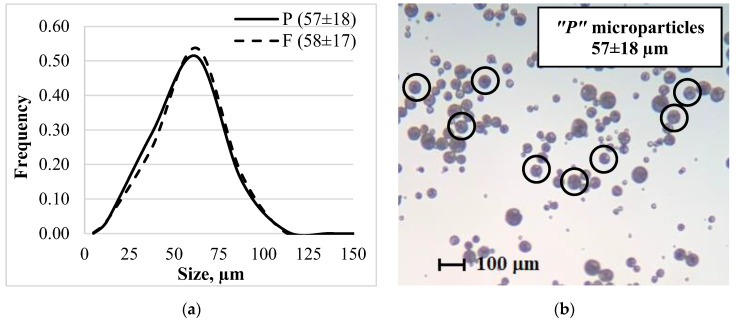
Microparticles properties: (**a**) Size distribution of the particles prepared with the single emulsion method; (**b**) Picture of the prepared microparticles.

The properties of the particles, such as size and size distribution, are shown in Figure 1. From the two different types of granules with the same production parameters, microparticles of the same particle size (57 ± 18 µm) could be produced. Mostly spherical particles that did not aggregate formed during the emulsion method.

### 3.2. Microparticles Essential Oil Adsorption from Different Concentration Solutions

#### 3.2.1. Essential Oil Adsorption from Ethanol Solutions

**Figure 2:**
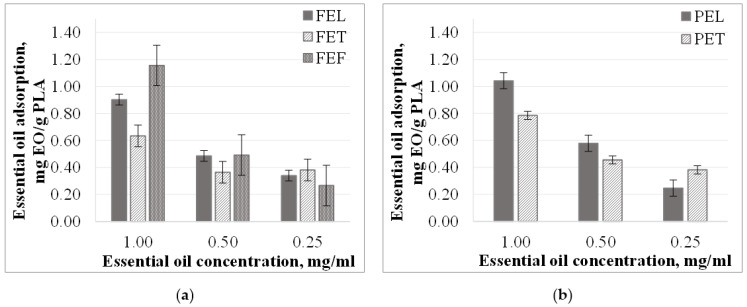
Essential oil adsorption (mg *EO*/g *PLA*) in ethanol solutions in the case of (**a**) particles from *F* granules; (**b**) particles from *P* granules.

For each sample, we concluded that the specific amount of essential oil adsorbed on the *PLA* surface or in its pores decreases with the decreasing concentration of essential oil (Figure 2). In 1.00 and 0.50 mg/mL concentration essential oil solutions, the specific amount of essential oil adsorbed in fennel essential oil solutions was the highest (1.16 and 0.49 mg *EO*/g *PLA*) followed by lemongrass (0.90 and 0.49 mg *EO*/g *PLA*), and thyme essential oil solutions (0.63 and 0.36 mg *EO*/g *PLA*). Exceptions were the particles tested in 0.25 mg/mL solution. In this case of the sorption in thyme essential oil solutions was the highest (0.38 mg *EO*/g *PLA*) followed by lemongrass and fennel essential oil solutions (0.34 mg *EO*/g *PLA* and 0.27 mg *EO*/g *PLA*). A similar correlation was observed for *P* samples. The correlation between specific essential oil uptake and essential oil solution concentration is close to linear (except for *FET* sample). The specific amount of the adsorbed essential oil did not change below a 0.5 mg/mL concentration in the case of the *FET* sample.

**Figure 3:**
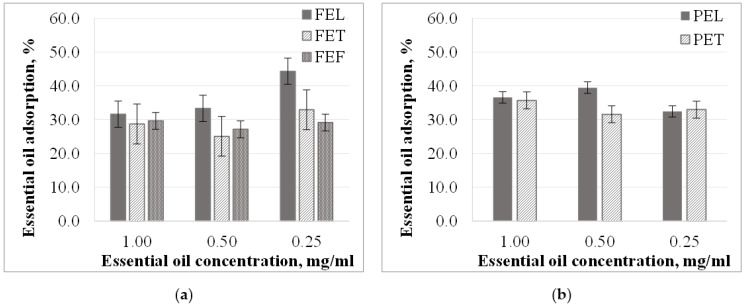
Essential oil adsorption (%) in ethanol solutions in the case of (**a**) particles from *F* granules; (**b**) particles from *P* granules.

Essential oil uptake of the particles prepared from the different *PLA* granules was different in ethanol solutions. There was ~10% difference in the amount of essential oil adsorbed by the particles. The *F* samples’ essential oil sorption was ~25–33%, while for the *P* samples it was ~30–40% (Figure 3). This may be due to the *PLA* content and the crystallinity degree of the granules being different. Adsorption is related to surface area through porosity. The crystallinity affects the structure (e.g., porosity) of the particles and thus their adsorption properties. Liu et. al. investigated the sorption properties of polystyrene (*PS*) and polyvinylchloride (*PVC*) with ciprofloxacin (*CIP*). They found that the sorption capacity of the microplastics decreased as the degree of crystallinity increased, whereas the opposite trend was observed after aging the microplastics. This indicates that crystallinity is not the main factor in determining the adsorption properties. [34] However, we found that the essential oil uptake of the more crystalline microparticles was on average 15% higher than that of the amorphous particles. This may be because the material system and the compatibility of the materials are different and, as a result, the adsorption properties are different. But crystallinity is not the only factor that can affect adsorption. The IR spectrophotometric method was used to determine that the *PLA* content of the granules *P* was 94%, and 89% for the other granules, while the crystallinity degrees were 12.4% and 1.0%, respectively. For this reason, there may also be a difference in the *PLA* content and the crystallinity of microparticles produced, causing the difference between adsorption properties.

The essential oil uptake did not change for the *FEF*, *FET*, and *PET* samples. The diversion was ±1.1%, ±3.2%, and ±1.7%. The exceptions were the *FEL* samples, where the essential oil uptake was inversely proportional to concentration. As the concentration decreases, the amount of essential oil adsorbed increases. No correlation could be found for the *PEL* samples.

In general, we concluded that essential oil uptake is mainly caused by differences between microparticles (degree of crystallinity, composition) rather than by the type of essential oils present in the solutions. However, the specific amount of essential oil adsorbed on the surface or in the pores of *PLA* was not the same for each essential oil, and decreased with decreasing essential oil concentration.

#### 3.2.2. Essential Oil Adsorption from Methanol Solutions

**Figure 4:**
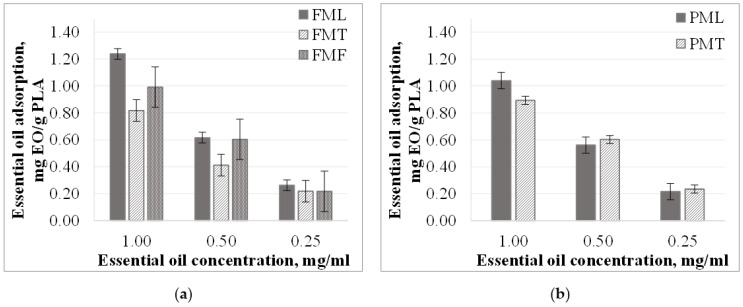
Essential oil adsorption (mg *EO*/g *PLA*) in methanol solutions in the case of (**a**) particles from *F* granules; (**b**) particles from *P* granules.

The specific amount of essential oil adsorbed was found to decrease with decreasing concentration of essential oil in methanol. (Figure 4). The relation between specific essential oil recordings and essential oil solution concentration is close to linear (except for *FMT* and *PMT* samples). In the case of *F* samples, the specific amount of essential oil adsorbed in lemongrass essential oil solutions was the highest, followed by fennel and thyme essential oil solutions. A similar correlation was observed for the other kinds of particles in 1.00 mg/mL concentration solutions. At lower concentrations, however, the specific amount of essential oil adsorbed was about the same (difference 0.02% and 0.01%). 

The essential oil adsorption of the particles prepared from two different *PLA* granules was also different in methanol solutions, just like in ethanol. For thyme essential oil, the essential oil sorption of the *F* samples was 10% lower than in the case of the *P* samples, while for lemongrass essential oil the essential oil uptake was higher for the *F* samples with the same amount. 

**Figure 5:**
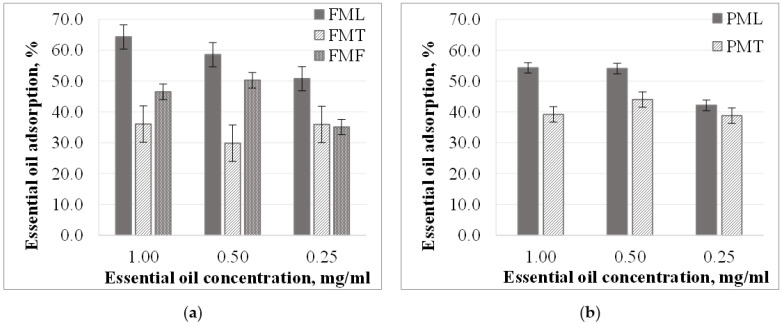
Essential oil adsorption (%) in methanol solutions in the case of (**a**) particles from *F* granules; (**b**) particles from *P* granules.

The essential oil uptake did not change significantly in *FMT* and *PMT* samples (Figure 5), and the diversion was 2.9–2.4%. In the case of *FML* samples, the essential oil uptake was proportional to concentration. As the concentration of the essential oil solution decreases, the amount of essential oil adsorbed decreases. For *FMF* and *PML* samples in 1.00 and 0.50 mg/mL concentration essential oil solutions, the essential oil uptake did not change (diversion 1.9% and 0.1%, respectively).

In contrast with ethanol solutions, when using methanol solvent, we found that essential oil uptake is affected not only by the properties of the particles but also by the type of essential oil itself. The amount adsorbed was about 23–10% higher for lemongrass essential oil than for thyme essential oil in methanol solutions. This difference was less than 8% in ethanol solutions.

### 3.3. Thermal Characteristic of the PLA Particles

Differential scanning calorimetry (*DSC*) was performed in order to know the thermal characteristics of the samples. We investigated how the thermal properties of the prepared microparticles differed from each other, and how the essential oil uptake affected the properties. The glass transition (*T_g_*), cold crystallization (*T_cc_*), and melting temperature (*T_m_*) of the *PLA* granules were determined from the second heating of the *DSC* measurement. The degree of crystallinity (*X_c_%)* was calculated from the melting enthalpy (Δ*H_m_*) and the cold crystallization enthalpy (Δ*H_cc_*), considering an ideal melting enthalpy (Δ*H_m_*^0^) of 94 kJ/kg [35,36].

*X_c_%* = [(Δ*H_m_* − Δ*H_cc_*)/Δ*H_m_*^0^] × 100, (1)

**Figure 6:**
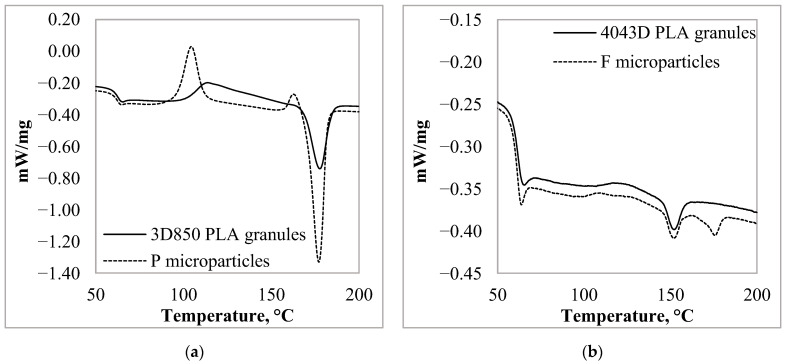
*PLA* granules and microparticles second heating DSC curves in the case of (**a**) 3D850 granules; (**b**) 4043D granules.

The *P* granules exhibited an exothermic, cold crystallization peak at 114 °C and an endothermic, melting peak at 177.6 °C (Figure 6a). The *F* granules also exhibited two peaks like *P* granules, but with lower *T_m_* (152.0 °C) and peak intensity (Figure 6b). The *F* granulate is amorphous with a low crystallinity degree (1.0%) while the *P* granulate is more crystalline with a 12.4% crystallinity degree.

In the case of the microparticles, the thermal characteristic of the *PLA* was different. For the *F* microparticles, the *T_g_* did not change (60.1 °C), but two melting peaks appeared at 151.7 °C and 175.5 °C. The *F* microparticles’ crystallinity decreased by 0.9%. Two cold crystallization peaks appeared in the case of the *P* microparticles. One peak at 104.6 °C and one just before the melting peak at 162.6 °C. The melting peak appeared at the same temperature (177 °C), but the change in the heat flow was greater. Accordingly, the degree of crystallinity increased to 16.1%.

**Figure 7:**
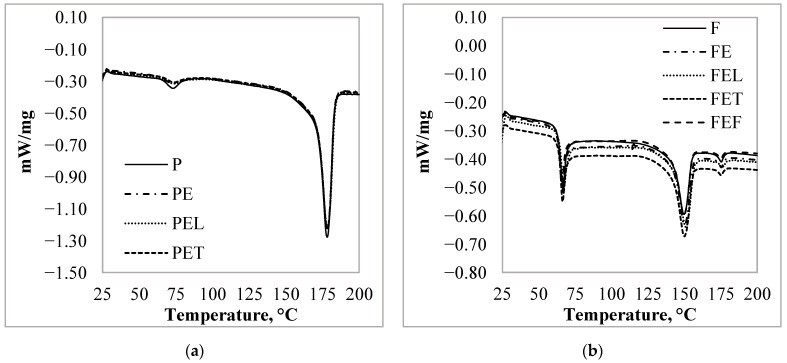
*PLA* microparticles first heating DSC curves after the adsorption measurement: (**a**) 3D850 granules; (**b**) 4043D granules.

To investigate the effect of essential oil on the thermal properties of the particles, we have analyzed the results of both the first and second heating. During the first heating melting peaks appeared. The glass transition and the melting peak of the *P*, *PE*, *PEC*, *PET,* and *PEF* samples appeared at the same temperature (Figure 7a), respectively 66.5 ± 0.3 °C, and 176.6 ± 0.2 °C. The degree of crystallinity was 56.8 ± 0.4%. For the other particle, each sample exhibited two melting peaks at 150.2 ± 0.6 °C and 175.4 ± 0.4 °C. The crystallinity degree of the *F* sample was 17.2% before the adsorption measurement. After the adsorption measurement, the degree of crystallinity of the particles increased to 19.07%.

**Figure 8:**
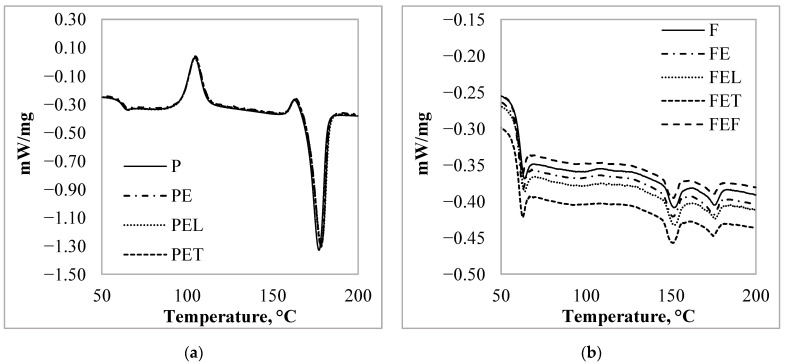
*PLA* microparticles second heating DSC curves after the adsorption measurement: (**a**) 3D850 granules; (**b**) 4043D granules.

During the second heating of the glass transition, the two cold crystallization peaks and the melting peak of the *PE*, *PEC*, *PET,* and *PEF* samples appeared at the same temperature as (Figure 8a) the *P* respectively 61.5 ± 0.5 °C, 104.5 ± 0.3 °C, 163.0 ± 0.4 °C, and 178.0 ± 0.8 °C. The degree of crystallinity was 17.3 ± 2.0%. For the *FE*, *FEC*, *FET,* and *FEF* samples, a glass transition also appeared at 59.4 ± 0.5 °C, a cold crystallization peak at 117.0 ± 0.3 °C, and two melting peaks at 151.4 ± 0.5 °C and 175.3 ± 0.8 °C (Figure 8b) like the reference, *F* sample.

Based on the results of the first and second heating, we concluded that after the adsorption measurement the thermal characteristic of the *P* microparticle did not change under the influence of the solvent or the essential oil solutions. However, the results of the first heating step for the *F* particles showed that the adsorption of essential oils had little effect on the properties of the *PLA*, as the degree of crystallinity of the particles increased by 2%.

As mentioned earlier, the two different types of granules have been developed for different applications, and consequently have different thermal properties in addition to their composition. About the composition, we know that the *PLA* content of the granules *P* was 94% and for the other granules it was 89%. For this reason, there may also be a difference in the *PLA* content and the crystallinity of microparticles produced, causing the difference between the thermal characteristic of the *PLA* particles. Based on the measurement results, we concluded that the thermal properties of the particles were not changed by the adsorption. 

Essential oils can act as a plasticizer in polymers. The plasticizer diffuses into the *PLA* polymer matrix and physically interacts with the polymer chains, resulting in an increase in free volume and a decrease in *T_g_*. Kamarudinthe et. al. investigated the effect of the incorporation of epoxidized jatropha oil (*EJO*) as a plasticizer and kenaf fiber on the thermal properties of *PLA*/*Kenaf*/*EJO* biocomposites. They found that the incorporation of *EJO* plasticizer at various concentrations did not result in any new peak or major shift of the existing peaks of the *PLA* composite. The *T_g_* was gradually decreased with the addition of treated kenaf and an increase in *EJO* loading [37]. Considering their results, we concluded that in our measurements, the amount of adsorbed essential oils was not sufficient to force a significant change in *PLA* structure.

### 3.4. Correlation of the Adsorption Properties with the Hansen Solubility Parameter

The solubility parameter and its components for each essential oil and solvent are given in Table 2. The essential oils’ solubility parameter was determined by calculation using the Hoftyzer–Van Krevele method [24,25,33]. The solubility of a material is largely determined by its chemical nature, so the solubility parameters of a component can also be calculated from its molecular structure. According to the Hoftyzer and Van Krevelen method, the solubility parameter components can be calculated with the following equations [33,38]:

*δ_d_* = (∑*F_di_*)/*V*, (2)

*δ_p_* = [∑(*F_pi_*)^2^]^1/2^/*V*, (3)

*δ_h_* = [∑*E_hi_*/*V*]^1/2^, (4)

where *F_di_* is the group contributions to the dispersion component (*F_d_*) of the molar attraction constant, *F_pi_* is the group contributions to the polar component (*F_p_*) of the molar attraction constant, E_hi_ is the hydrogen bonding energy of the structural group and *V* the molar volume. The *F_di_*, *F_pi_*_,_ and *E_hi_* of the structural group derived from group contribution tables. We estimated solubility parameters for the main components of the essential oils [38].

There is a difference in the composition of the essential oil solutions, which is characterized by the solubility parameter and its components. Accordingly, we can determine how the change in properties (for example the polarity) caused by different compositions of the essential oils affects the adsorption properties of *PLA* particles.

The affinity between the polymer and the component can be characterized by the *RED* (relative energy difference). If the *RED* < 1 the polymer is soluble in the solvent, if *RED* > 1, it is not soluble in the solvent. Based on the solubility parameter and the *RED* (Table 2), the polymer–solvent affinity smaller in the case of methanol than ethanol. The RED for methanol is higher (1.64) than that for ethanol (1.33). In the case of essential oils, the solubility is highest in the case of lemongrass essential oil (with 0.65 *RED*), followed by thyme (with 0.89 *RED*) and fennel (with 2.39 *RED*) essential oils.

**Figure 9:**
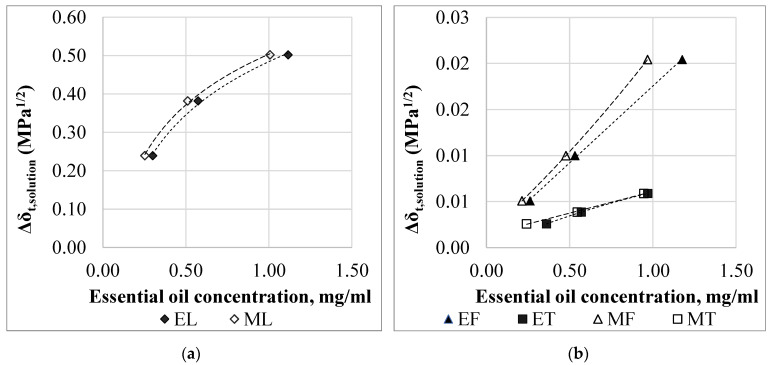
The change of the *HSP* parameters depending on the essential oil concentration in the case of (**a**) lemongrass essential oil; (**b**) thyme and fennel essential oils.

In the given concentration range (0.25–1.00 mg/mL) as the concentration of the essential oil solutions increases, the total solubility parameter (*δ_t,solution_*) of the solutions decreases. The higher the essential oil concentration, the greater the change in the solution total solubility parameter (Δ*δ_t,solution_*), which is the difference between the solvent solubility parameter and the essential oil solubility parameter (Figure 9). The lower the solubility parameter, the better the solubility of the polymer. Accordingly, the solutions with the highest essential oil concentrations were expected to have the highest essential oil uptake, which was confirmed by the measurement results.

For thyme and fennel essential oils solutions, the essential oil does not significantly affect the solvents’ solubility parameters (Figure 9). For thyme and fennel essential oils, the change in *δ_t,solution_* is two orders of magnitude smaller compared to the lemongrass essential oil.

**Figure 10:**
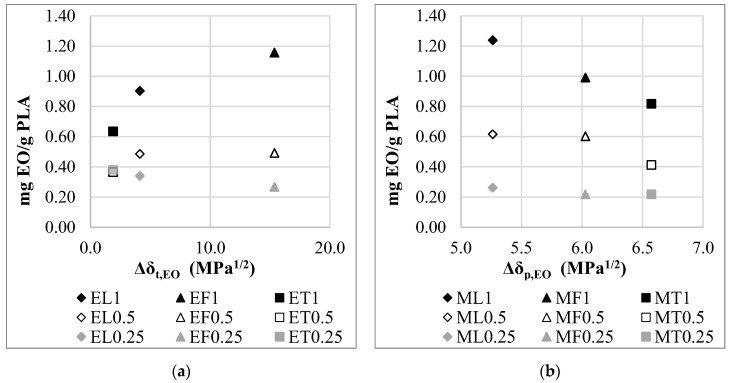
Correlation between the *HSP* parameters and the specific essential oil uptake in the case of (**a**) ethanol solutions; (**b**) methanol solutions.

The specific amount of essential oil adsorbed on the *PLA* can be also related to the *HSP* and its components. Essential oils of different compositions have different solubility parameters depending on their composition (Table 2). Consequently, the specific essential oil uptake of *PLA* is different for different essential oils. In ethanol solution, we found a correlation between the adsorbed amount of the essential oil and Δ*δ_t_*_,*EO*_. The Δ*δ_t_*_,*EO*_ shows how much the total solubility parameter of the *EO* differs from the solubility parameter value for *PLA*. In 1.00 mg/mL and 0.50 mg/mL concentration solutions, as the value of the Δ*δ_t_*_,*EO*_ increases, the amount of adsorbed essential oil increases (Figure 10a). The opposite effect was found in the case of the 0.25 mg/mL concentration solution: as the value of the Δ*δ_t_*_,*EO*_ increases, the amount of adsorbed essential oil slightly decreases. However, in methanol solutions, the amount of adsorbed specific essential oil can be associated with the *δ_p_* component of the *HSP* parameter, which indicates polar interactions. In 1.00 mg/mL and 0.50 mg/mL concentration solutions, the *δ_p_* value of the essential oil is closer to the *δ_p_* value of the *PLA*, the greater the specific amount of adsorbed essential oil increases (Figure 10b).

We found that while the specific amount of essential oil adsorbed in methanol solutions is mainly affected by the polarity of the essential oils, in ethanol solutions the total solubility parameter affects the adsorption. As expected, it was confirmed that the higher the concentration of the solution, the greater the effect of the essential oil properties (polarity and solubility) on the adsorption. Not just the polarity of the components, but also the hydrogen bonding parameter can determine the properties of the polymer. In contrast to our finding, Sato et al. found that the *δ_h_* is more determinant in the solubility properties of the polymer than the other two solubility parameter components (*δ_d_* and *δ_p_*) [25].

## 4. Conclusions

In our study, we investigated a method for the determination of the adsorption properties of different polylactic acid particles for different additives like essential oils. We investigated the effect of the essential oil concentration (0.25 mg/mL, 0.50 mg/mL, and 1.00 mg/mL) on the *PLA* particles’ sorption properties. We used two types of polymer granules (NatureWorks Ingeo 4043D and 3D850) with different degrees of crystallinity (1.0% and 12.4% respectively) and different *PLA*-content (89% and 94% polylactic acid content) to prepare the microparticles with the solvent evaporation emulsification method. We prepared the suitable particles to carry out the test. 

We obtained different result for the essential oil adsorption of the particles prepared from two different *PLA* granules. We concluded that in ethanol solutions the essential oil uptake is mainly caused by differences between microparticles’ properties (degree of crystallinity, composition) rather than by the type of essential oils present in the solutions. However, in methanol solutions, the type of the essential oil and its properties also affect the essential oil uptake. The specific essential oil uptake of the more crystalline microparticles (*X_C_%* = 16.1%) was on average 15% higher than that of the amorphous particle (*X_C_%* = 0.1%). For all samples, we concluded that the specific amount of essential oil adsorbed decreases (with average 38–53%) with the decreasing concentration (0.25–1.00 mg/mL) of essential oil in both ethanol and methanol solutions. The particles showed the highest essential oil adsorption capacity in the methanol solution of lemongrass essential oil. It was higher than 50%.

The reason for the difference between the adsorption of the essential oils is that there is a difference in the composition of the essential oil solutions, which is well characterized by the *HSP*. We found that while the specific amount of essential oil adsorbed in methanol solutions is mainly affected by the polarity of the essential oils, in ethanol solutions the total solubility parameter affects the adsorption. These correlations may be suitable for model-based prediction of the polymer adsorption properties. By further investigation of changes in physical properties, the resulting data stores can be used to support application and processing. 

## Figures and Tables

**Table 1:** Notation for the samples.

Particles Type		Solvents		Essential Oils		Solution Concentration	
*P*	from 3D850 *PLA*	*E*	Ethanol	*L*	Lemongrass essential oil	*0.25*	0.25 mg/mL
*F*	from 4043D *PLA*	*M*	Methanol	*T*	Thyme essential oil	*0.5*	0.5 mg/mL
				*F*	Fennel essential oil	*1*	1 mg/mL

**Table 2:** Solubility parameters and *RED* values of essential oils, solvents, and the *PLA*.

Material	*δ_d_*, (J/cm)^1/2^	*δ_p_*, (J/cm)^1/2^	*δ_h_*, (J/cm)^1/2^	*δ_t_*, (J/cm)^1/2^	*RED*
*PLA*	18.6	9.9	6.0	21.9	-
Lemongrass essential oil	16.4	4.6	5.1	17.8	0.65
Thyme essential oil	21.3	3.2	9.2	23.8	0.89
Fennel essential oil	24.3	3.9	28.0	37.3	2.39
Ethanol	15.1	8.4	18.3	25.2	1.33
Methanol	14.5	11.5	21.4	28.3	1.64

## Data Availability

The data presented in this study are available on request from the corresponding author.
